# Combination of graduated compression stockings and intermittent pneumatic compression is better in preventing deep venous thrombosis than graduated compression stockings alone for patients following gynecological surgery: a meta-analysis

**DOI:** 10.1186/s12959-024-00636-1

**Published:** 2024-07-12

**Authors:** Limei Lu, Ya Shen, Yuping Pan

**Affiliations:** 1 Department of Gynaecology, Huzhou Maternity & Child Health Care Hospital, Huzhou, 313000 Zhejiang China; 2 Nursing Department, Huzhou Maternity & Child Health Care Hospital, No 2 East Road, Wuxing District, Huzhou, 313000 Zhejiang China; 3 Health Education Division, Huzhou Maternity & Child Health Care Hospital, Huzhou, 313000 Zhejiang China

**Keywords:** Deep venous thrombosis, Compression stockings, Intermittent pneumatic compression device

## Abstract

**Background:**

Deep vein thrombosis (DVT) is common in patients undergoing gynecological surgery. We aimed to investigate the preventive efficacy in DVT of graduated compression stockings (GCS) alone and in combination with intermittent pneumatic compression (GCS + IPC) after gynecological surgery.

**Methods:**

In November 2022, studies on the use of GCS and GCS + IPC for the prevention of DVT after gynecological surgery were searched in seven databases. After literature screening and data extraction based on specific inclusion and exclusion criteria, preventive efficacies, including the risk of DVT and anticoagulation function, of GCS and GCS + IPC were compared. Finally, sensitivity analysis and Egger’s test were performed to evaluate the stability of the meta-analysis.

**Results:**

Six publications with moderate quality were included in this meta-analysis. The results showed that GCS + IPC significantly reduced DVT risk (*P* = 0.0002) and D-dimer levels (*P* = 0.0005) compared with GCS alone. Sensitivity analysis and Egger’s test showed that the combined results of this study were stable and reliable.

**Conclusions:**

Compared with GCS alone, GCS + IPS showed a higher preventive efficacy against DVT in patients following gynecological surgery.

**Supplementary Information:**

The online version contains supplementary material available at 10.1186/s12959-024-00636-1.

## Highlights

GCS + IPC could significantly reduce the risk of DVT than GCS alone.

GCS + IPC could significantly reduce the D-dimer than GCS alone.

The involved studies were all RCTs and had no significant publication bias.

Sensitivity analysis indicated that the combined results were stable.

**Supplementary Information:**

The online version contains supplementary material available at 10.1186/s12959-024-00636-1.

## Background

Venous thromboembolism (VTE) is one of the most common cardiovascular disorders, affecting 5% of patients. VTE mainly manifests as lower extremity deep venous thrombosis (DVT) or pulmonary embolism (PE) [1]. DVT is a serious postoperative complication following gynecological surgery [2, 3]. The incidence of DVT after surgery for gynecological malignancies or other gynecological surgeries ranges from 15 to 45% [4]. DVT manifests as swelling and pain in the lower extremities (leg and pelvic veins) [5]. In severe cases, the thrombus can move from the legs to the lungs, causing PE and leading to rapid respiratory and circulatory disorders, and life-threatening conditions [6, 7]. Therefore, prevention of DVT is crucial in clinical practice.

The clinical prevention methods for DVT include drug administration and mechanical prophylaxis [8]. Drug treatment, especially heparin, commonly poses a risk of bleeding [8]. Therefore, it is of great practical significance to explore methods for preventing DVT in addition to drug treatment. Mechanical prevention methods include graduated compression stockings (GCS) and intermittent pneumatic compression (IPC). GCS can prevent thrombus formation in the legs by providing varying amounts of pressure to different parts of the legs [7]. IPC can prevent thrombosis by increasing venous blood flow and decreasing stasis in leg veins [9]. In a previous report, the combination of GCS and IPC (GCS + IPC) was found to be better than GCS alone in preventing VTE after gynecological surgery [4]. However, Wand et al. reported that there were no significant differences in GCS + IPC or GCS for the prevention of VTE after gynecological surgery [6]. The reason for this inconsistency may be the heterogeneity of the patients and the limitations of the data. Moreover, studies with larger sample sizes are absent. Hence, there is an urgent need to obtain a more comprehensive result on the effectiveness of GCS and GCS + IPC in the prevention of DVT.

Meta-analyses provide a general and effective understanding of many inconsistent studies [10]. In this study, we investigated the differences in postoperative DVT and coagulation indices between GCS and GCS + IPC treatments in patients following gynecological surgery to better understand the prophylactic effectiveness of these options.

## Method

### Search strategy

A search strategy was established for publications in PubMed, Embase, Cochrane Library, Web of Science, Wanfang Data, China National Knowledge Infrastructure, and the China Science and Technology Journal Database databases. The search terms were “graduated compression stockings,” “venous thrombosis,” “deep venous thrombosis,” “prothrombin time,” “activated partial thromboplastin time,” “thrombin time,” “d-dimer,” “fibrinogen,” and “platelet.” Terms in the same category were combined with “OR” and those in different category were combined with “AND.” Database-specific and free-text terms were combined for the search, and the search formula was adjusted based on the characteristics of the databases. The detailed retrieval procedures are listed in Table S1-4. The search was conducted until November 29, 2022. No language restrictions were imposed. Moreover, to obtain more publications for meta-analysis, we screened relevant reviews and references.

### References selection

The inclusion criteria were as follows: (1) the participants were gynecological surgery patients; (2) the groups included GCS + IPC and GCS; (3) the study type was a randomized controlled trial (RCT); (4) one or more of the following outcomes was reported: DVT, prothrombin time (PT), activated partial thromboplastin time (APTT), thrombin time (TT), D-dimer, fibrinogen (FIB), platelet count (PLT).

The exclusion criteria were as follows: (1) non-original articles such as reviews, conference abstracts, and comments were excluded; (2) studies on non-gynecological surgery patients were excluded; and (3) when the same data was used in multiple publications, the one with the most comprehensive information was included, while the others were excluded.

### Data extraction and quality assessment

Two investigators reviewed publications according to these criteria. After determining the literature included in the meta-analysis, data extraction was performed according to a prepared table. The information to be extracted included the name of the first author, publication year, basic characteristics of the study subjects (sample size and age), number and diagnostic method of DVT, and coagulation indicators (PLT, PT, APTT, D-dimer, FIB, and TT). After completing the above extraction, any disagreement between the two investigators was discussed and a consensus was reached. The quality of the RCTs was assessed using the Cochrane Collaboration tool [11].

### Statistical analysis

Differences of discrete (DVT) and continuous (coagulation indicators) variables were compared using risk ratio (RR) and weighted mean difference (WMD), respectively. Heterogeneity among the studies was assessed using Cochran’s Q test and I^2^ statistics [12]. Q test *P* < 0.05 or I^2^ > 50% was considered as significant heterogeneity, and a random effects model was used for meta-analysis. *P* ≥ 0.05 and I^2^ ≤ 50% were recognized as non-significant heterogeneity and a fixed effect model was used for meta-analysis. The effects of a single study on the meta-analysis results were evaluated by removing one study at a time [13]. Publication bias among the studies was evaluated using funnel plots and Egger tests [14]. All statistical analyses were performed using RevMan 5.3 and Stata12.0 software.

## Results

### Results of references selection

**Fig. 1:**
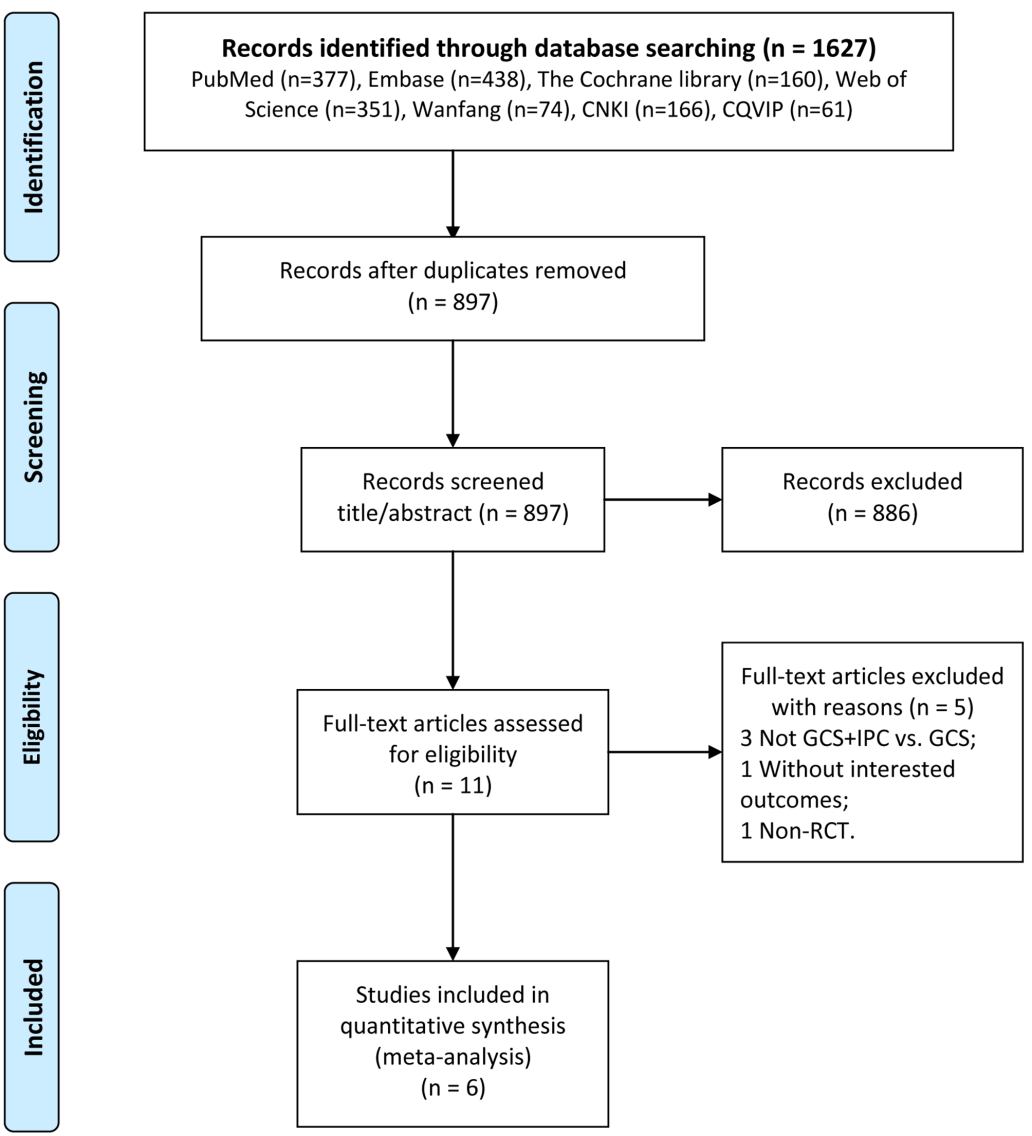
Process and results of the publication screening. CNKI, China National Knowledge Infrastructure; CQVIP, China Science and Technology Journal Database; GCS, graduated compression stockings; IPC, intermittent pneumatic compression

The process and results of this study are shown in Fig. 1. A total of 1627 publications were retrieved from public databases. After excluding 730 publications, 897 articles were retained. We then checked the titles and abstracts and found that 886 articles did not meet the inclusion criteria. In the selected 11 publications, five were excluded after reading the full text, among which three did not analyze GCS + IPC vs. GCS, one was without the outcomes of interest, and one was not an RCT study. Finally, six publications [4, 6, 15–18] were included in the meta-analysis.

### Characteristics of the six publications included

The six studies were all conducted in China, and all patients were diagnosed with postoperative DVT using ultrasound (Table 1). The sample sizes of the six studies were between 60 and 312 for a total of 980. A total of 485 and 495 patients were treated with GCS + IPC and GCS, respectively. No significant differences in age or surgical method were observed between the GCS + IPC and GCS alone groups.

**Table 1:** Characteristics of six included studies in this meta-analysis

Study	Type of surgery	Groups	*n*	Age, years	Route of surgery (LA/LS/VS)	Outcomes
Gao, J 2012	GPS	GCS + IPC	52	60.89 ± 11.64	10/32/10	DVT, PT, TT, APTT, D-dimer
		GCS	56	59.38 ± 10.16	17/28/11	
Li, XJ 2014	GPS	GCS + IPC	75	49.62 (41–73)	49/38/63	DVT, PT, APTT, PLT, FIB
		GCS	75			
Lin, XL 2010	Malignant tumor	GCS + IPC	135	54.2 (43–78)	NR	DVT, PT, APTT
		GCS	135			
Sang, CQ 2018	GPS	GCS + IPC	153	52.6 ± 9.9	59/94/0	DVT
		GCS	159	54.2 ± 9.4	59/100/0	
Wang, L 2017	GPS	GCS + IPC	30	56.59 (45–77)	NR	DVT, PT, TT, APTT, D-dimer, PLT, FIB
		GCS	30			
Xu, H 2020	GPS	GCS + IPC	40	49.11 ± 11.85	1/39/0	DVT
		GCS	40	48.27 ± 12.01	1/39/0	

DVT, deep vein thrombosis; GCS, graduated compression stockings; GPS, Gynaecological pelvic surgery; IPC, intermittent pneumatic compression; LA/LS/VS, Laparotomy/Laparoscopic surgery/Vaginal surgery; PT, prothrombin time; APTT, activated partial thromboplastin time; TT, thrombin time; FIB, fibrinogen, PLT, platelet count

### Results of quality assessment

As shown in Figure S1, there was no blinding information in the six original studies, and most of the studies lacked information on random sequence generation and allocation concealment. Overall, the quality of the included studies was moderate.

### Differences in the risk of DVT between the GCS + IPC and GCS groups

**Fig. 2:**
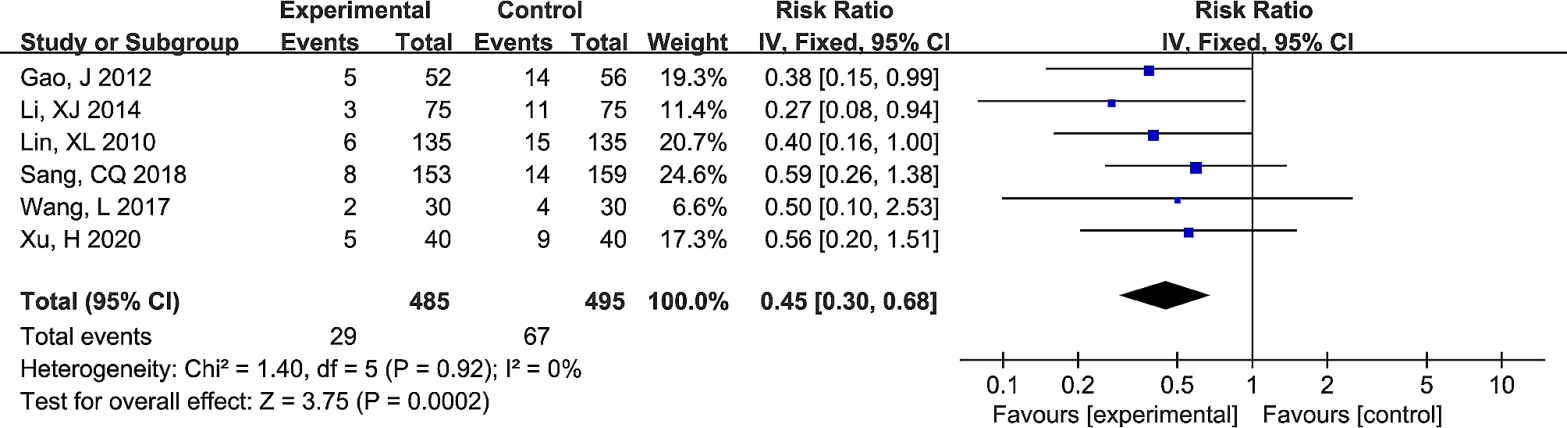
Forest plot of differences in the risk of DVT between the GCS + IPC and GCS groups. DVT, deep venous thrombosis

The differences in the risk of DVT between the GCS + IPC and GCS groups are shown in Fig. 2. Heterogeneity test results showed I^2^ = 0% and *P* = 0.92; therefore, we generated data using a fixed-effects model. The results showed that significant differences were observed between the GCS + IPC and GCS groups (RR (95%CI) = 0.45 (0.30, 0.68); *P* = 0.0002). Compared to GCS alone, the risk of postoperative DVT decreased significantly in gynecological surgery patients in the GCS + IPC group.

### Differences in coagulation indicators between the GCS + IPC and GCS groups

**Fig. 3:**
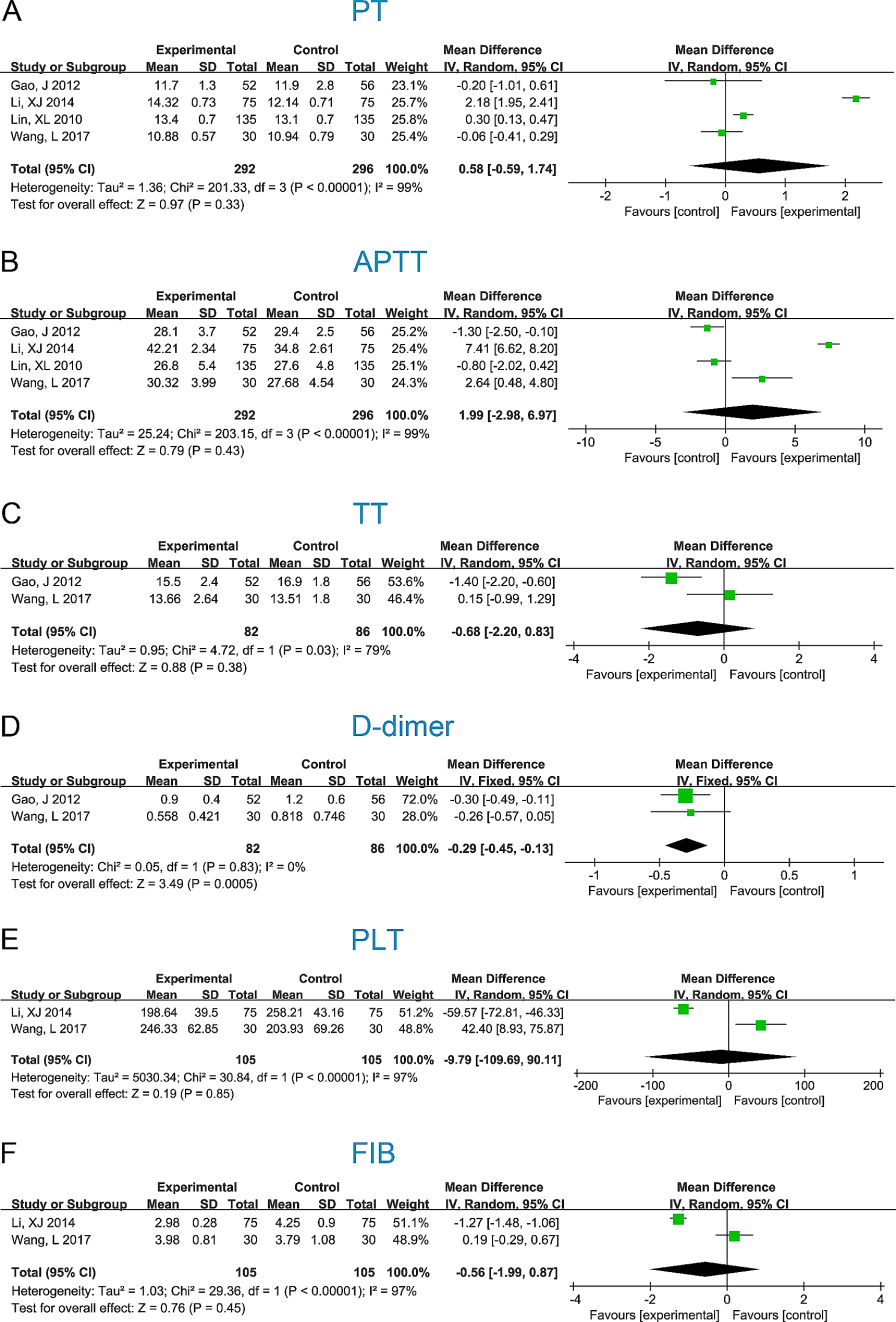
Forest plots of differences in coagulation function between GCS + IPC and GCS groups. PT, prothrombin time; APTT, activated partial thromboplastin time; TT, thrombin time; FIB, fibrinogen; PLT, platelet count

The coagulation indicators (PT, APTT, TT, D-dimer, PLT, and FIB) were compared between the GCS + IPC and GCS groups (Fig. 3). Two studies reported the D-dimer outcomes and showed no significant heterogeneity (I^2^ = 0%, *P* = 0.83). Therefore, we combined the results using a fixed-effects model. The combined results showed significant differences between the GCS + IPC and GCS groups (WMD [95%CI] = -0.29 [-0.45, -0.13], *P* = 0.0005); the GCS + IPC group possessed lower D-dimer levels than the GCS group. For the other five indicators, significant heterogeneity was observed (I^2^ > 50%, *P* < 0.05), and the combined results showed no statistical significance (*P* < 0.05).

### Sensitivity analyses and publication bias

**Fig. 4:**
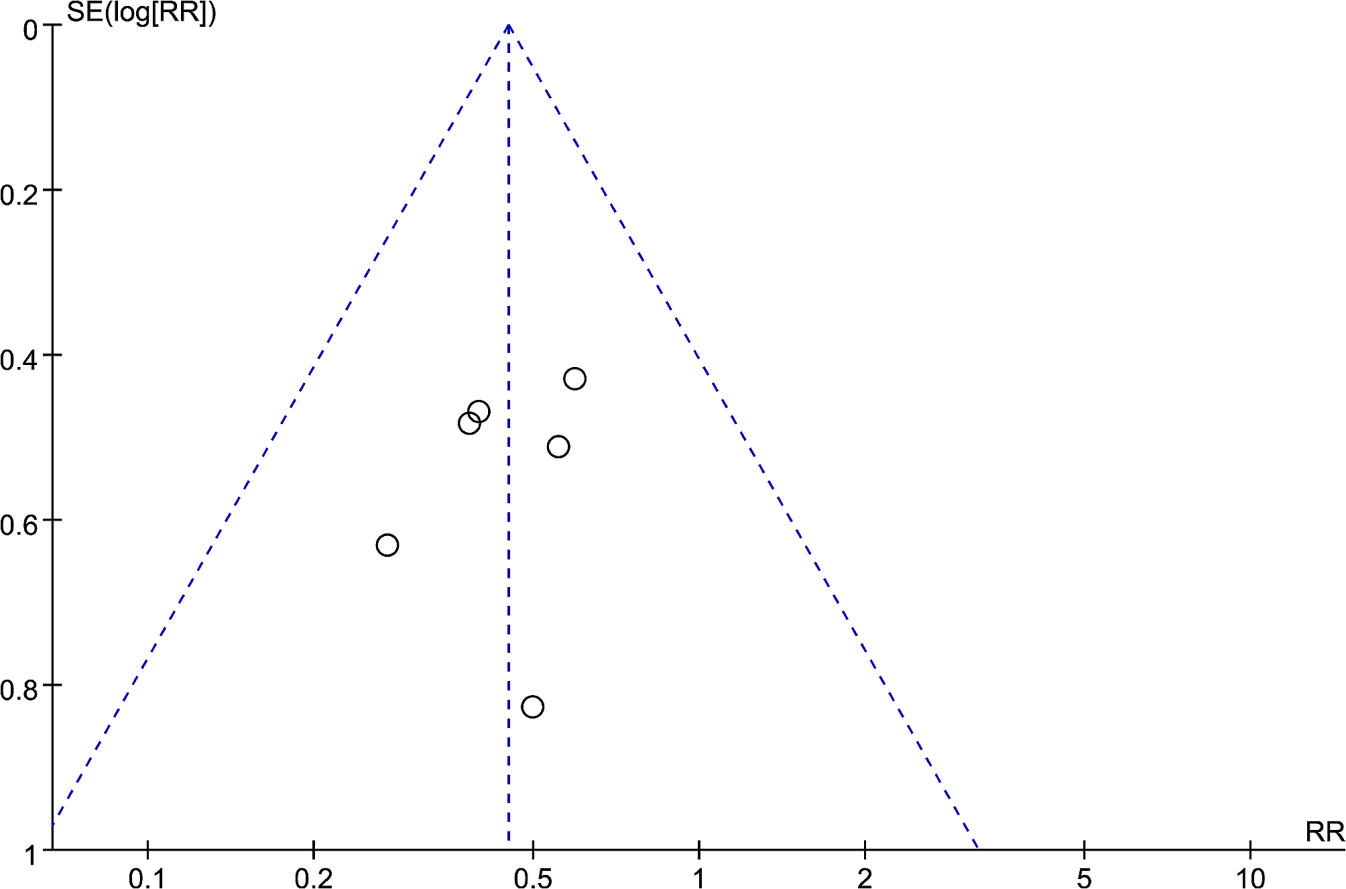
Funnel plot showing the distribution of the studies

Considering that the analyses for indicators were based on fewer than five studies, we only conducted sensitivity and publication bias analyses for DVT. After removing one study at a time, the combination for the remaining studies still showed significant differences between GCS + IPC and GCS groups, whose RR (95%CI) changed from 0.43 (0.27, 0.68) to 0.48 (0.31, 0.75) and *P* values were less than 0.05. These results indicated that the meta-analysis in our study was stable. The Egger test showed *P* = 0.529, and the funnel plot exhibited good symmetry in the distribution of scatter points (studies) (Fig. 4). Both the quantitative and qualitative analyses revealed no significant publication bias for DVT.

## Discussion

After gynecological surgery, lower limb swelling, congestion, and pain are inevitable, and the incidence of DVT is high. Many risk factors such as varicose veins, age > 50 years, D-dimer > 0.5 mg/L, hypertension, operative time ≥ 60 min, bedrest days > 3 days, as well as intraoperative pneumoperitoneum pressure ≥ 15 mmHg are associated with DVT development in patients with gynecological surgery [19, 20], hinting that taking counteractive or preventive measures specific to these factors may help decrease the incidence of DVT after gynecological surgery.

GCS and IPC are the main mechanical prevention strategies for DVT after gynecological surgery [7, 9]. Many studies have used GCS + IPC to obtain a better preventive effect against DVT. In current studies, whether GCS + IPC exhibits a better preventive effect than GCS alone is inconsistent [4, 6]. Therefore, in the present study, we compared the efficacy of GCS alone and GCS + IPC in preventing DVT and improving coagulation function in patients after gynecologic surgery. Our results indicate that GCS + IPC could reduce the risk of DVT and elevated D-dimer levels in patients after gynecologic surgery.

Mechanical prophylaxis aims to reduce venous stasis, which increases the risk of postoperative VTE. Venous stasis in the legs reduces blood flow and the pulsatile index, which can increase the risk of DVT. GCS and IPC can reduce venous stasis using passive and active methods, respectively. Using GCS alone can reduce 50% of DVT formation [21]. Some studies have also found that IPC devices are as effective as pharmaceutical prevention in reducing DVT intraoperatively and postoperatively after major gynecologic surgery [22, 23]. Moreover, the efficacy of GCS can be significantly improved by combining it with other prevention methods [21], indicating a cumulative effect of GCS when combined with other methods. This hypothesis was confirmed by our results that GCS + IPC could significantly reduce the risk of DVT and D-dimer levels compared with that obtained with GCS alone. D-dimer is a degradation product of soluble fibrin that results from the breakdown of thrombi [24]. Decreased D-dimer levels indicate lower coagulation function and DVT risk.

In this study, we demonstrated that GCS + IPC significantly reduced the risk of DVT. The included studies were all RCTs, and the risk of bias, such as loss to follow-up and reporting bias, was small. Moreover, no significant publication bias was observed, indicating the reliability of the results. The sensitivity analysis also demonstrated the stability of the combined results. However, this study has some limitations. First, some indicators (such as PT, APTT, TT, PLT, and FIB) exhibited significant heterogeneity, and the number of included studies was limited. The source of heterogeneity could not be explored using subgroup analysis or meta-regression. Second, the included studies in this meta-analysis were all from China, which significantly limits the generalizability of the results owing to disparities in thromboembolism prevalence among different ethnicities [25, 26]. Extrapolation of the results should be performed with caution. Third, the number of included studies was small. Lastly, patients who undergo surgery for gynecologic malignancies are at a heightened risk of developing DVT, which is attributable to factors including older age, cancer type, presence of genetic mutations predisposing to clot formation, endothelial damage from pelvic lymphadenectomy, extended surgical procedures, and thrombogenic effects of certain chemotherapy drugs [27, 28]. However, the included studies did not report relevant data on the occurrence of DVT postoperatively in patients with malignant or benign diseases, which hindered the comparison of their respective risks. Collectively, more high-quality, large sample RCTs are needed to further verify the stability and generalizability of these results.

## Conclusion

Compared with preventive treatment with GCS alone, GCS + IPS showed higher preventive efficacy for DVT in patients following gynecological surgery.

### Electronic supplementary material

Below is the link to the electronic supplementary material.

Supplementary Material 1

Supplementary Material 2

## Data Availability

No datasets were generated or analysed during the current study.

## Abbreviations

APTT: Activated partial thromboplastin time
DVT: Deep vein thrombosis
FIB: Fibrinogen
GCS: Graduated compression stockings
IPC: intermittent pneumatic compression
PE: pulmonary embolism
PLT: platelet count
PT: prothrombin time
RR: risk ratio
TT: thrombin time
VTE: venous thromboembolism
WMD: weighted mean difference
