# Supplementary figures and images for: Combination of graduated compression stockings and intermittent pneumatic compression is better in preventing deep venous thrombosis than graduated compression stockings alone for patients following gynecological surgery: a meta-analysis
Source: Thromb J. 2024 Jul 12;22:63. doi: 10.1186/s12959-024-00636-1 (PMC11245769; doi:10.1186/s12959-024-00636-1)

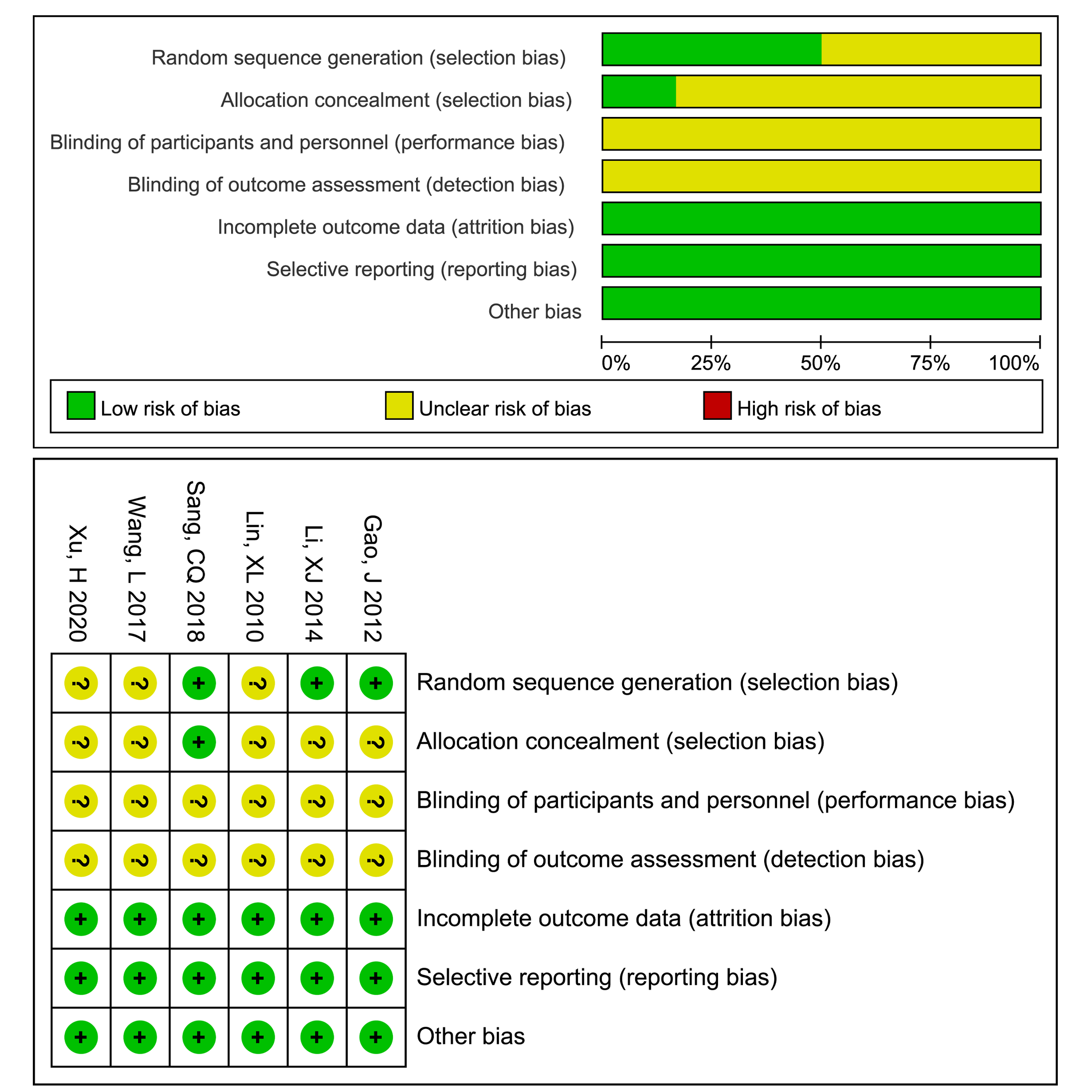

Supplement: Supplementary file 1 — Supplementary Material 1 [file 12959_2024_636_MOESM1_ESM.tif]
