# Supplementary material for: Combination of graduated compression stockings and intermittent pneumatic compression is better in preventing deep venous thrombosis than graduated compression stockings alone for patients following gynecological surgery: a meta-analysis
Source: Thromb J. 2024 Jul 12;22:63. doi: 10.1186/s12959-024-00636-1 (PMC11245769; doi:10.1186/s12959-024-00636-1)
Supplement: Supplementary file 2 — Supplementary Material 2 [file 12959_2024_636_MOESM2_ESM.docx]

The retrieval time: 20221129

**Table S1 The results of retrieval in PubMed**

| Search | Query | Items found |
| --- | --- | --- |
| #1 | ("graduate"[All Fields] OR "graduated"[All Fields] OR "graduates"[All Fields]) AND ("stockings, compression"[MeSH Terms] OR "compression stockings"[All Fields]) | 529 |
| #2 | "venous thrombosis"[MeSH Terms] OR "venous thrombosis"[All Fields] | 74536 |
| #3 | "deep venous thrombosis"[All Fields] OR "DVT"[All Fields] OR "deep vein thrombosis"[All Fields] | 33375 |
| #4 | "venous thromboembolism"[MeSH Terms] OR "venous thromboembolism"[All Fields] | 29721 |
| #5 | ("prothrombin time"[MeSH Terms] OR "prothrombin time"[All Fields]) OR ("partial thromboplastin time"[MeSH Terms] OR "activated partial thromboplastin time"[All Fields]) OR ("thrombin time"[MeSH Terms] OR "thrombin time"[All Fields]) OR ("fibrin fragment d"[Supplementary Concept] OR "fibrin fragment d"[All Fields] OR "d dimer"[All Fields]) OR ("fibrinogen"[MeSH Terms] OR "fibrinogen"[All Fields] OR "fibrinogens"[All Fields]) OR ("blood platelets"[MeSH Terms] OR "platelet"[All Fields] OR "platelets"[All Fields]) | 384605 |
| #6 | #2 OR #3 OR #4 OR #5 | 475147 |
| #7 | #1 AND #6 | 377 |

**Table S2 The results of retrieval in Embase**

| Search | Query | Items found |
| --- | --- | --- |
| #1 | graduated AND ('compression stockings'/exp OR 'compression stockings') | 586 |
| #2 | ('venous thrombosis'/exp OR 'venous thrombosis') | 148671 |
| #3 | ('deep venous thrombosis'/exp OR 'deep venous thrombosis' OR dvt OR 'deep vein thrombosis'/exp OR 'deep vein thrombosis') | 88330 |
| #4 | ('venous thromboembolism'/exp OR 'venous thromboembolism') | 181858 |
| #5 | ('prothrombin time'/exp OR 'prothrombin time' OR 'activated partial thromboplastin time'/exp OR 'activated partial thromboplastin time' OR 'thrombin time'/exp OR 'thrombin time' OR 'd-dimer'/exp OR 'd-dimer' OR 'fibrinogen'/exp OR 'fibrinogen' OR 'platelet'/exp OR 'platelet' OR 'platelets') | 559511 |
| #6 | #2 OR #3 OR #4 OR #5 | 773179 |
| #7 | #1 AND #6 | 438 |

**Table S3 The results of retrieval in Web of Science**

| Search | Query | Items found |
| --- | --- | --- |
| #1 | graduated compression stockings (All Fields) | 502 |
| #2 | (venous thrombosis) OR (deep venous thrombosis) OR DVT OR (deep vein thrombosis) OR (venous thromboembolism) (All Fields) | 78439 |
| #3 | ("prothrombin time" OR "activated partial thromboplastin time" OR "thrombin time" OR "d dimer" OR "fibrinogen" OR "platelet" OR "platelets") (All Fields) | 249957 |
| #4 | #2 OR #3 | 316430 |
| #5 | #1 AND #4 | 351 |

**Table S4 The results of retrieval in the Cochrane library的检索步骤和结果**

| Search | Query | Items found |
| --- | --- | --- |
| #1 | MeSH descriptor: [Stockings, Compression] explode all trees | 274 |
| #2 | (compression stockings):ti,ab,kw (Word variations have been searched) | 1164 |
| #3 | #1 OR #2 | 1164 |
| #4 | (graduated):ti,ab,kw (Word variations have been searched) | 3693 |
| #5 | #3 AND #4 | 251 |
| #6 | MeSH descriptor: [Venous Thrombosis] explode all trees | 2859 |
| #7 | MeSH descriptor: [Venous Thromboembolism] explode all trees | 805 |
| #8 | ((venous thrombosis) OR (deep venous thrombosis) OR DVT OR (deep vein thrombosis) OR (venous thromboembolism)):ti,ab,kw (Word variations have been searched) | 12555 |
| #9 | #6 OR #7 OR #8 | 13271 |
| #10 | MeSH descriptor: [Venous Thromboembolism] explode all trees | 813 |
| #11 | MeSH descriptor: [Partial Thromboplastin Time] explode all trees | 505 |
| #12 | MeSH descriptor: [Thrombin Time] explode all trees | 46 |
| #13 | MeSH descriptor: [Fibrinogen] explode all trees | 1767 |
| #14 | MeSH descriptor: [Blood Platelets] explode all trees | 2043 |
| #15 | ((prothrombin time) OR (activated partial thromboplastin time) OR (thrombin time) OR (d-dimer) OR (fibrinogen OR platelet OR platelets)):ti,ab,kw (Word variations have been searched) | 40705 |
| #16 | #10 OR #11 OR #12 OR #13 OR #14 OR #15 | 41428 |
| #17 | #9 OR #16 | 52056 |
| #18 | #5 AND #17 | 172 |
| #19 | #10 in Trials | 160 |
